# Early efficacy of postoperative rehabilitation training for lumbar disc herniation

**DOI:** 10.1186/s13102-023-00704-5

**Published:** 2023-08-08

**Authors:** Yuwei Zhu, Shuang Xia, Weihang Yang, Fengchao Shi, Hongjian Ji

**Affiliations:** 1 Department of Orthopedics, The Sixth Affiliated Hospital of Nantong University, Yancheng Third people’s Hospital, Yancheng, Jiangsu 224001 China; 2 Jiangsu Vocational College of Medicine, Yancheng, Jiangsu 224005 China

**Keywords:** Lumbar disc herniation, Postoperative rehabilitation training, Prognostic factors

## Abstract

**Objective:**

To investigate the early clinical efficacy of rehabilitation training after unilateral biportal endoscopy for lumbar disc herniation and to analyze the prognostic factors.

**Methods:**

A total of 100 patients with lumbar disc herniation who underwent unilateral biportal endoscopy at The Sixth Affiliated Hospital of Nantong University from January 2019 to January 2021 were retrospectively analyzed. The control group was given a standard home-based exercise program, while the intervention group was given a substituted rehabilitation training opposed to a standard home-based exercise program. The early postoperative pain relief and quality of life values were compared between the two groups, and the independent risk factors affecting the prognosis of patients were analyzed.

**Results:**

There were no significant differences in sex, age, smoking, drinking, BMI, course of disease, type of disc herniation, preoperative VAS, ODI or SF-36 between the two groups (*P* > 0.05). There was no significant difference in preoperative and postoperative VAS and ODI scores at 3 months between the two groups (*P* > 0.05), yet there were significant differences in postoperative VAS and ODI at 12 months (*P* < 0.05). The SF-36 score of the intervention group was lower than that of the control group at 12 months, and the difference was statistically significant (*P* < 0.05). The excellent rate of the Macnab standard modification used in the intervention group was 88.00% at 12 months, and that in the control group was 62.00%. The difference between the two groups was considered to indicate a statistically significant (*P* < 0.05). The results of logistic multivariate regression model analysis showed that rehabilitation training (95% CI: 1.360–12.122, *P* = 0.012), the type of intervertebral disc (95% CI: 0.010–0.676, *P* = 0.020), and age (95% CI: 1.056–8.244, *P* = 0.039) were independent risk factors affecting the prognosis of patients.

**Conclusion:**

Postoperative rehabilitation training can effectively relieve pain and improve quality of life; thus, it is highly recommended in the clinic. Postoperative rehabilitation training, intervertebral disc type and age are independent risk factors for the postoperative prognosis of lumbar intervertebral disc herniation.

## Introduction

Lumbar disc herniation (LDH) is a common and frequently occurring disease in clinics, with an incidence of 2%-3% [1]. The main symptom is low back pain combined with radiculopathy [2], which can lead to disability [3]. Studies have shown a higher prevalence in male patients aged 45–64. The incidence of herniation to L4/5 and L5/S1 is higher than that of the other segments, which exceeds 90% [4]. As society continues to develop and changes in people's working styles and living habits increase, the incidence of LDH is on the rise, advancing early in the youth ages, which seriously affects the quality of life and health of patients [5].

The current treatment of LDH mainly includes conservative treatment and surgical treatment. Approximately 87% of patients can be relieved with conservative treatment [6]. Surgical treatment is usually considered for those who fail to respond to conservative treatment and who suffer from persistent lumbar and leg pain or progressive aggravation of neurological dysfunction [7]. As a new spinal endoscopy technique, unilateral biportal endoscopy (UBE) is widely used in the clinic due to the advantages of double channels. It establishes an endoscope portal and instrument portal in the posterior approach of the spine, which the surgeon can perform under continuous irrigation of normal saline. PAO et al. [8] applied UBE technology to lumbar spinal stenosis. Postoperative VAS, ODI, and JOA were significantly improved, and postoperative MRI examination showed that the dural sac area increased from 71.4 ± 36.5 mm^2^ to 177.3 ± 59.2 mm^2^. Postoperative CT examination of the lumbar spine showed that the retention rate of facet joints was 84.2% on the approach side and 92.9% on the contralateral side. The preservation of the facet joint to the greatest extent ensures the stability of the spine after surgery. It has been recognized by surgeons for its advantages, such as minimal trauma, flexible operation, clear vision and thorough decompression [9–11]. However, due to the need to peel off the paravertebral muscle in spinal surgery, the distal nerve of the muscle is innervated, which may lead to intractable back pain after surgery. At the same time, the weakening of the muscles in the lower back will cause instability in the lumbar spine [12, 13].

In the past, postoperative rehabilitation training has been recognized by many scholars [14–16]. However, the study showed that patients who were referred for rehabilitation training after routine surgery for lumbar disc herniation showed no significant changes in postoperative pain relief, improvement in function and quality of life compared to patients who were not referred [17, 18]. This study was conducted to investigate the early clinical efficacy of rehabilitation training after unilateral biportal endoscopy for lumbar disc herniation and to analyze the prognostic factors.

## Materials and methods

### General clinical data

**Fig. 1:**
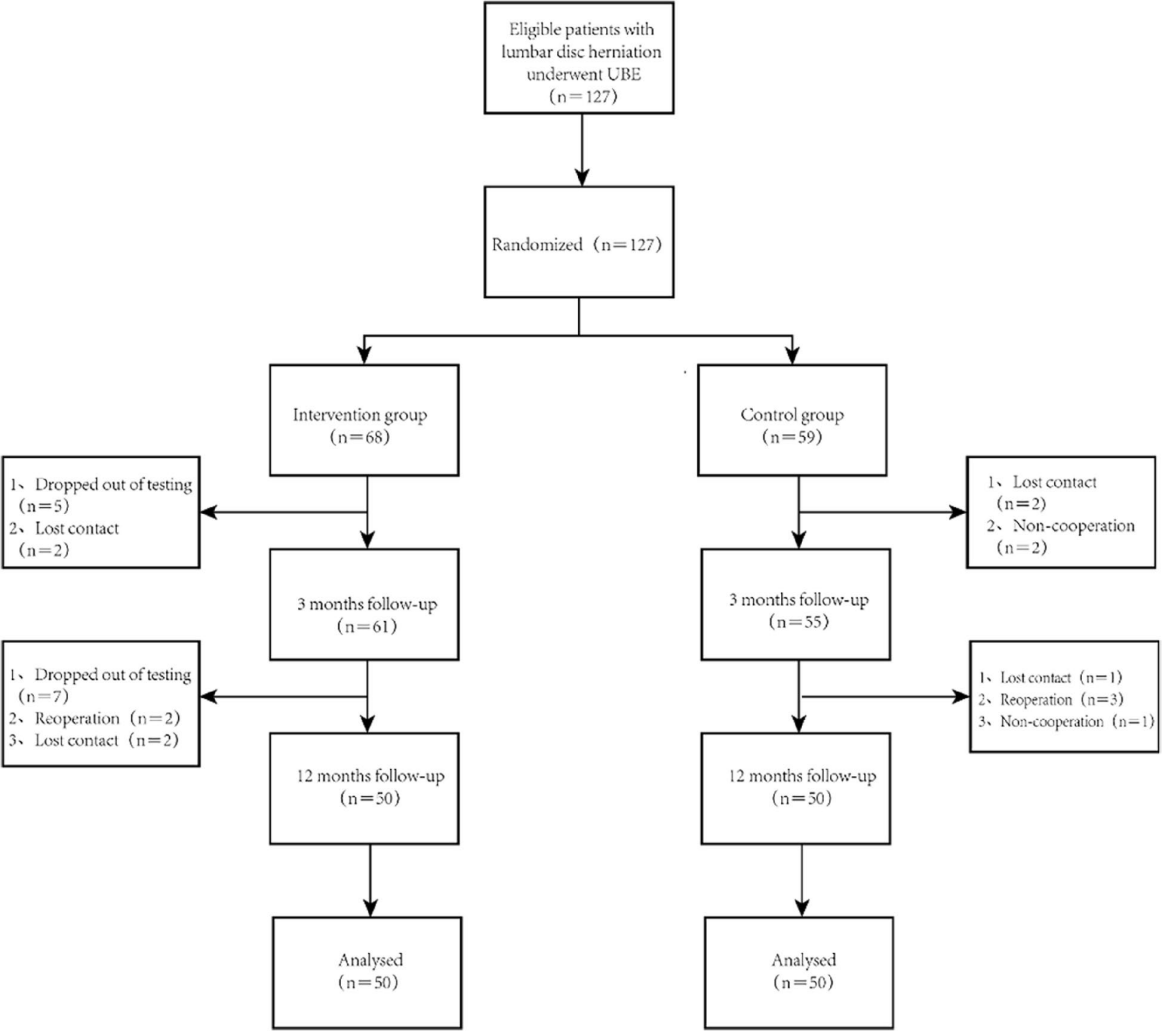
Follow-up procedure

A total of 100 patients with lumbar disc herniation were included, including 50 in the intervention group and 50 in the control group (Fig. 1). This study was approved by the Medical Ethics Committee of The Sixth Affiliated Hospital of Nantong University, and informed consent was obtained from patients and their families.

### Inclusion and exclusion criteria

Inclusion criteria: ① Single-level lumbar disc herniation was confirmed by CT, MRI and other imaging examinations. ② Patients with severe symptoms of nerve compression and ineffective conservative treatment for 6 months. ③Good cardiopulmonary function and no operation contraindications.

The exclusion criteria were as follows: ① Previous history of lumbar surgery; ② Other spinal diseases (such as spinal tuberculosis); and ③ Severe lumbar instability.

### Functional exercise methods

The control group received postoperative health education, and patients were instructed to perform a standard home-based exercise program after discharge, including hip and knee extension, straight leg raise exercise, walking and stretching of the waist.

**Fig. 2:**
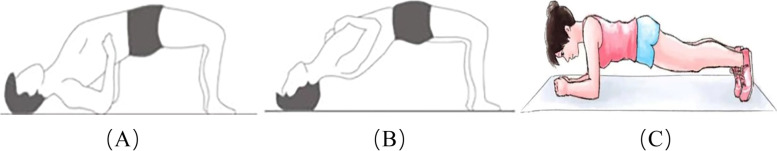
Five-point support, three-point support and Planck diagram

The intervention group received a standard home-based exercise program and postoperative rehabilitation training. Rehabilitation training is mainly for lumbar core muscle training. Lumbar core muscle training is an effective intervention method to control spinal instability by strengthening the strength of core stable muscles through a series of targeted trainings [19]. The details were as follows: (1) within 1 day after waking up from anesthesia, the ankle pump was performed actively on the bed, and the straight leg elevation test was performed passively; (2) 1–7 days after surgery, the straight leg raising test was performed actively, and the abdominal brace was worn to actively get out of bed and perform exercises such as hip extension, knee extension and hip flexor, and the walking distance was slowly increased; (3) One week after surgery, the following training was gradually carried out:① Five-point support: lying in the supine position on the exercise pad, bending of the hips and knees, the feet were as wide as the hips, and the bilateral upper arms were attached to the ground, with the feet, elbows, and back shoulders as support; Exhale the waist with the buttocks slowly raised to the highest point, and hold for 3–5 s, and then inhale the waist with buttocks slowly down to the starting position, up and down for a time, 20–30 times/group, repeat 3 groups, the interval between Groups 1 min (Fig. 2 A). ② Three-point support: When adapted to the five-point support practice, you can try to switch to the three-point support. Cross your hands in front of your torso, using your feet and back shoulders as support points, using the same five-point support (Fig. 2 B). ③ Plank: positioned prone on the exercise pad, elbows perpendicular to the ground, feet on the ground, trunk straight, head, shoulders, hips and ankles are steady to remain balanced in the plane position, eyes to the ground, maintain uniform breathing, 30–60 s/group, 2–4 groups/time, group rest for 1 min (Fig. 2 C). ④ Lying on the back in the supine position on the exercise pad, slowly flexing the left knee so that the thigh is close to the chest. Place your arms around your thighs or knees and pull them toward your chest. Hold for 10 s and repeat with the other leg. ⑤ Lie on your back on the exercise pad, bend your hips and knees, hold your knees with both hands, and slowly raise your knees. Lift your knees to your chest and hold for approximately 10 s, then repeat. ⑥ Take a standing position with your feet shoulder width apart and step back with your right foot. Bend your left knee and bring your weight back to your right hip. While keeping the right leg at a right angle, bend further forward and down to the right leg, extending the outside of the hip joint. Repeat with the other leg. Under the direction of the tube bed doctor and under the supervision of the tube bed nurse during hospitalization. After discharge, under the supervision of the patient's family members, photos of daily punched rehabilitation training were chatted through a WeChat group, and the doctor in charge of the bed asked about postoperative rehabilitation training through phone calls and WeChat every week.

### Observation indicators

The visual analog scale (VAS) and Oswestry Disability Index (ODI) were compared at 3 months and 12 months postoperatively, and the SF-36 quality of life scale and modified Macnab criteria were compared at 12 months. The follow-up was mainly carried out by outpatient review and telephone consultation.

### Analysis of related factors

Among the 100 LDH patients who underwent surgical treatment, 75 patients with excellent and good Macnab criteria were taken as the effective group, and the other 25 patients were taken as the ineffective group, and the postoperative efficacy was taken as the independent variable. At the same time, the relevant indicators of the two groups, including sex, age, rehabilitation training, smoking, drinking, BMI, course of disease, and type of disc herniation, were counted as the dependent variables for univariate analysis. Factors with *P* < 0.05 were taken as the dependent variable for multivariate logistic regression model analysis.

### Statistical analysis

SPSS26.0 software was used for data processing. Quantitative data with VAS, ODI and SF-36 scores conforming to a normal distribution were expressed as the mean ± standard deviation. Independent sample t tests were used for intergroup comparisons, and paired t tests were used for intragroup comparisons. Analysis of variance was used to compare VAS and ODI scores at different time points of the sample. The count data were expressed as percentages, and the chi-square test was used. A multiple logistic regression model was used to predict risk factors. *P* < 0.05 was considered statistically significant.

## Results

### Basic data

In the intervention group, there were 24 males and 26 females, with an average age of 53.58 ± 12.70, 25 smokers, 25 nonsmokers, 35 drinkers, 15 nondrinkers, 24 patients with BMI < 30 and 26 patients with BMI ≥ 30, with an average disease course of 10.86 ± 3.95, 33 patients with intervertebral disc herniation and 17 patients with intervertebral disc prolapse. In the control group, there were 30 males and 20 females, with an average age of 55.30 ± 14.09, 22 smokers, 2 nonsmokers, 30 drinkers, 20 nondrinkers, 32 patients with BMI < 30 and 18 patients with BMI ≥ 30, with an average course of 11.64 ± 2.88, 40 patients with intervertebral disc herniation and 10 patients with intervertebral disc herniation. There was no significant difference in basic data between the two groups (*P* > 0.05) (Table 1).

**Table 1:** Comparison of basic data between the two groups

	Intervention group (*N* = 50)	Control group (*N* = 50)	t/*x*^2^	*P*
Sex (Male/Female)	24(48.00%) /26(52.00%)	30(60.00%) /20(40.00%)	1.449	0.229
Age	53.58 ± 12.70	55.30 ± 14.09	-0.641	0.523
Smoke (Yes/No)	25(50.00%) /25(50.00%)	22(44.00%) /28(56.00%)	0.361	0.548
Drink (Yes/No)	35(70.00%) /15(30.00%)	30(60.00%) /20(40.00%)	1.099	0.295
BMI (< 30/ ≥ 30)	24(48.00%) /26(52.00%)	32(64.00%) /18(36.00%)	2.597	0.107
Course	10.86 ± 3.95	11.64 ± 2.88	-1.128	0.262
Type (Herniated/prolapse)	33(66.00%) /17(34.00%)	40(80.00%)/10(20.00%)	2.486	0.115

### Degree of pain and modified Macnab standard

Postoperative VAS and ODI scores within the two groups were significantly decreased at 3 months and 12 months, and the difference was statistically significant (*P* < 0.05). There was no significant difference in preoperative and postoperative VAS and ODI scores at 3 months between the two groups (*P* > 0.05), but there were significant differences in postoperative VAS and ODI at 12 months (*P* < 0.05). The postoperative modified Macnab standard at 12 months showed that in the intervention group, 22 cases were excellent, 22 cases were good, 6 cases were fair, and the rate of excellent and good was 88%. In the control group, 10 cases were excellent, 21 cases were good, 13 cases were fair, and 6 cases were poor, and the rate of excellent and good was 62%. The difference between the two groups was indicated as statistically significant (*P* < 0.05) (Table 2).

**Table 2:** Comparison of preoperative and postoperative VAS, ODI and postoperative modified Macnab criteria between the two groups

	Intervention group	Control group	t/*x*^*2*^	*P*
Preoperative VAS (point)	7.36 ± 0.83	7.66 ± 0.87	-1.766	0.081
3 months postoperative VAS (point)	2.54 ± 0.58	2.74 ± 0.75	-1.492	0.139
12 months postoperative VAS (point)	1.94 ± 0.62	2.62 ± 1.23	-3.498	0.001
Preoperative ODI(%)	66.72 ± 6.35	67.98 ± 6.11	-1.011	0.315
3 months postoperative ODI(%)	20.28 ± 5.13	21.48 ± 6.41	-1.034	0.304
12 months postoperative ODI(%)	17.08 ± 5.93	21.90 ± 10.20	-2.888	0.005
12 months postoperative modified Macnab criteria	44/50(88%)	31/50(62%)	9.013	0.003

One-way analysis of variance was performed in the VAS and ODI groups at different time points, and the result was *P* < 0.05

### Quality of life

The scores of postoperative physiological function, physiological function, physical pain, health status, energy, social function, emotional function and mental health within the two groups were significantly improved at 12 months, and the differences were considered statistically significant (*P* < 0.05). There was no significant difference in preoperative scores between the two groups (*P* > 0.05). The scores of the intervention group were higher than those of the control group at 12 months postoperatively, and the differences were statistically significant (*P* < 0.05) (Table 3).

**Table 3:** Comparison of SF-36 Life scale scores preoperatively and postoperatively between the two groups

	Preoperative		t	*P*	Postoperative		t	*P*
	Intervention group	Control group			Intervention group	Control group		
PF	33.98 ± 5.53	33.80 ± 6.67	0.147	0.883	64.30 ± 5.89	59.80 ± 9.74	2.796	0.006
RP	37.00 ± 12.62	35.00 ± 12.37	0.800	0.425	71.00 ± 11.69	59.00 ± 18.04	3.947	< 0.01
BP	30.85 ± 9.05	31.38 ± 9.09	0.292	0.771	71.88 ± 9.88	66.24 ± 15.69	2.151	0.034
GH	47.30 ± 7.30	46.80 ± 6.76	0.355	0.723	72.90 ± 8.40	63.02 ± 10.91	5.074	< 0.01
VT	40.30 ± 5.38	39.10 ± 5.22	1.132	0.261	71.02 ± 12.43	62.50 ± 14.19	3.194	0.002
SF	51.25 ± 7.25	49.25 ± 8.15	1.296	0.198	75.50 ± 10.40	69.50 ± 14.76	2.350	0.021
RE	47.29 ± 16.60	51.95 ± 16.70	1.400	0.165	86.64 ± 16.53	71.96 ± 20.62	3.929	< 0.01
MH	52.72 ± 7.03	51.76 ± 8.10	0.633	0.528	79.84 ± 6.51	76.16 ± 8.81	2.374	0.020

*PF* Physical Functioning, *RP* Role-Physical, *BP* Bodily Pain, *GH* General Health, *VT* Vitality, *SF* Social Functioning, *RE* Role-Emotional, *MH* Mental Health

A paired sample T test was performed before and after the operation in the same group, *P* < 0.05

### Multifactor logistic regression analysis results

With postoperative efficacy as the dependent variable and sex, age, rehabilitation training, smoking, drinking, BMI, course of disease and type of disc herniation as independent variables, univariate analysis showed that age, rehabilitation training and type of disc herniation had statistical significance (*P* < 0.05), which were factors affecting the postoperative prognosis of patients with lumbar disc herniation (Table 4). The indicators with statistically significant differences in univariate analysis (age, rehabilitation training, type of disc herniation) were taken as independent variables, and the postoperative efficacy was taken as the dependent variable. The results of logistic multivariate analysis showed that rehabilitation training (95% CI: 1.360–12.122, *P* = 0.012), type of intervertebral disc (95% CI: 0.010–0.676, *P* = 0.020) and age (95% CI: 1.056–8.244, *P* = 0.039) were independent risk factors affecting the prognosis of patients (Table 5).

**Table 4:** Univariate analysis of influencing postoperative efficacy

	Effective group (*N* = 75)		Ineffective group (*N* = 25)		*x*^*2*^	*P*
	Patients	Incidence(%)	Patients	Incidence(%)		
Sex					2.630	0.105
Male	37	49.33	17	68.00		
Female	38	50.67	8	32.00		
Age					5.556	0.018
< 60	50	66.67	10	40.00		
≥ 60	25	33.33	15	60.00		
Rehabilitation					9.013	0.003
Yes	44	58.67	6	24.00		
No	31	41.33	19	76.00		
Smoke					1.084	0.298
Yes	33	44.00	14	56.00		
No	42	56.00	11	44.00		
Drink					3.297	0.069
Yes	45	60.00	20	80.00		
No	30	40.00	5	20.00		
BMI					1.948	0.163
< 30	45	60.00	11	44.00		
≥ 30	30	40.00	14	56.00		
Course					0.656	0.418
< 12	37	49.33	10	40.00		
≥ 12	38	50.67	15	60.00		
Type					8.946	0.003
Herniated	49	65.33	24	96.00		
Prolapse	26	34.67	1	4.00

**Table 5:** Multivariate logistic regression analysis affecting postoperative outcome

Factors	β	Sβ	Wald	P	95% CI
Rehabilitation	1.401	0.558	6.307	0.012	1.360–12.122
Type	-2.502	1.077	5.399	0.020	0.010–0.676
Age	1.082	0.524	4.259	0.039	1.056–8.244

## Discussion

Patients with LDH are mainly characterized by chronic back and leg pain, and some patients have muscle weakness or hyporeflexia [20]. Chronic back pain usually leads to changes in the structure and function of paravertebral muscles [21]. The UBE technique can completely decompress the nerve roots, aiding in relieving symptoms [22]. However, due to muscle atrophy caused by intraoperative muscle dissection, trunk muscle strength decline and delayed spinal instability can occur [23], and the repair effect of surgery on muscle structure and function is poorly indicated. Studies have shown that the lack of functional exercise in muscles reduces the blood supply, which causes muscle glycogen to produce large amounts of lactic acid under hypoxic conditions, leading to the production of pain [24, 25]. The purpose of rehabilitation training is to increase the tolerance of muscles and tissues, avoid slow recovery due to muscle atrophy, and reduce the distress of patients. Therefore, to promote recovery in patients as soon as possible, postoperative functional exercise is needed to strengthen the lumbar muscles and to achieve the effect of lumbar stability [26].

This study showed that the VAS and ODI scores of the intervention group were significantly lower than those of the control group, indicating that rehabilitation training can improve blood circulation, prevent muscle atrophy, enhance muscle strength, increase the stability of the lumbar spine and significantly improve back pain in patients compared with standard home-based exercise programs. To further explore the functional recovery of LDH patients at the injury site, all patients were followed up for quality of life evaluations at 12 months postoperatively. The results showed that the quality of life in the intervention group was higher than that in the control group, and the improvement rate of modified Macnab at 12 months postoperatively was higher in the intervention group (88.00%) than in the control group (62.00%). It is suggested that rehabilitation training can effectively improve the quality of life of patients with lumbar disc herniation. However, in this study, there were 6 patients with poor postoperative prognosis, 6 of whom were over 60 years old, including 0 cases in the intervention group and 6 cases in the control group. In this study, we concluded through multivariate logistic regression model analysis that age was an independent risk factor affecting the prognosis of patients. This may be related to the lower water content of lumbar intervertebral discs in elderly patients [24]. We believe that the degree of disc herniation is a factor affecting the prognosis of patients. The larger the volume of disc herniation, the better the prognosis of patients. We believe that this is mainly due to the thorough decompression of the nerve root after the intraoperative use of forceps to remove the large amount of herniated disc tissue, which allows further release of the nerve root compared to the small volume of protrusion.

He et al. [27] carried out postoperative continuous care for patients with lumbar disc herniation through the WeChat platform, and the follow-up results showed that the improvement in postoperative quality of life, JOA score and ODI of patients with continuous care was more obvious than that of routine continuous care, which was consistent with the results of our study. This shows that continuous rehabilitation training can improve the quality of life and spinal function of patients with lumbar disc herniation after surgery. Schwartz et al. [28] conducted a prospective cohort study and suggested that exercise after spinal surgery can improve mental health and spinal recovery and suggested long-term exercise. Lyu et al. [29] proposed staged rehabilitation and integrated kinetic chain exercise such as lumbar, pelvic, and leg training based on McKenzie technology and core stabilization muscle exercise. The effect of this program is significantly better than that of conventional lumbar and back muscle exercise, and it also provides us with a choice for postoperative rehabilitation. This indicates that rehabilitation training can be carried out regularly at home once knowledgeable of the specific operation process for rehabilitation training.

In conclusion, early rehabilitation training after UBE can reduce the pain in the back and legs of patients, improve the quality of life, and promote the rehabilitation of patients with lumbar disc herniation. However, for older patients, postoperative rehabilitation in pain relief and quality of life improvement still needs further research. This study provides a certain reference for the clinical rehabilitation of lumbar disc herniation after surgery, but there are also some shortcomings: ① the sample size is not large enough, and the results may be biased; ② the follow-up time of this study is not long enough, which can only show the early efficacy of rehabilitation training; and ③ some patients may not fully cooperate with the training during the implementation of the study, which may affect the results of the study. Further research will consider an increase in the number of samples, and measure to increase patient cooperation and long-term follow-up.

## Data Availability

The datasets used and/or analyzed during the current study are available from the corresponding author on reasonable request.
